# Rapid increase in the body mass index of very preterm infants is a risk factor for iron deficiency during infancy

**DOI:** 10.1038/s41598-023-42531-1

**Published:** 2023-09-19

**Authors:** Hyun Ho Kim, Eun Jee Lee, Jin Kyu Kim

**Affiliations:** 1 Department of Pediatrics, Jeonbuk National University School of Medicine, Jeonju, South Korea; 2 Research Institute of Clinical Medicine of Jeonbuk National University-Biomedical Research Institute of Jeonbuk National University Hospital, Jeonju, South Korea; 3 Department of Statistics and Data Science, Korea National Open University, Seoul, South Korea; 4 College of Nursing, Research Institute of Nursing Science, Jeonbuk National University, Jeonju, South Korea; 5 Department of Pediatrics, Jeonbuk National University Children’s Hospital, 20 Geonjiro, Jeonju, 54907 South Korea

**Keywords:** Outcomes research, Paediatric research

## Abstract

Iron deficiency (ID) in very preterm infants born at 28–32 weeks of gestational age (GA) can lower mental and motor test scores. This study aimed to determine whether the rapid growth of very preterm infants might be associated with ID. Among 134 very preterm born between January 2014 and December 2020 at Jeonbuk National University Hospital and discharged home, 93 were included in this study. Rapid BMI increase (RBI) was defined as a z-score difference of > 1 standard deviation between birth and 8 months. ID occurred in 23 of 93 (24.7%) infants at 8 months of corrected age (CA). ID was more common in the RBI group (50%) than in the non-RBI group (18.7%). In the multivariate logistic regression corrected for GA, infants small for gestational age (SGA) (odds ratio [OR] 6.06, 95% confidence interval [CI] 1.34–30.21) and RBI by z-score (OR 4.26, 95% CI 1.28–14.65) were identified as independent risk factors for ID at 8 months of CA. Conclusively, both SGA and RBI in the early life of very preterm were risk factors for ID at 8 months of CA.

## Introduction

Iron deficiency (ID) remains a serious health problem in children, even if it is not accompanied by anemia. The prevalence of ID among newborns fluctuates considerably across different studies; however, it remains alarmingly high. For instance, a prospective cohort study conducted on very-low-birth-weight infants (VLBWI) in Brazil reported a prevalence of 48.0% at 1 year of corrected age (CA), whereas studies on European term infants aged 1–3 years reported a prevalence ranging from 5 to 20%^1,2^. Preterm infants are at risk of ID because of a combination of physiological factors such as lower storage iron concentrations at birth and shorter half-life of red blood cells. In neonates, ID can result in lower mental and motor test scores, and it can be prevented by iron supplementation^3^. Iron supplementation from 6 weeks to 6 months can reduce the risk of ID and ID anemia (IDA) in these infants, without short-term adverse effects on morbidity or growth^4^. Assessing the nutritional status of very preterm infants and iron testing during follow-up visits is important.

Very preterm infants born at 28–32 weeks of gestational age (GA) experience catch-up growth by 18–24 months of CA. Successful catch-up growth in very preterm infants positively affects neurocognitive long-term outcomes^5^. The optimal catch-up growth rate is unknown, except that the catch-up completion point is 18–24 months. However, rapid growth in very preterm infants, rather than skeletal maturity, tends to increase the risk of overweight status and obesity^6,7^. Moreover, whether excessively fast catch-up growth leads to good outcomes in very preterm infants is unclear because their postnatal growth rate is similar to their intrauterine growth rates. Thus, this study aimed to determine whether the rapid growth of very preterm infants is an ID-associated factor.

## Methods

This study included very preterm infants discharged from the neonatal intensive care unit (NICU) of JeonBuk National University Hospital (JBUH) between January 2014 and December 2020 based on the data collected from the electronic medical records. Patients who were diagnosed with hemoglobinopathy and/or were not followed-up at 8 months of CA and those without available data on physical examination findings, diet, and blood test results on the medical chart were excluded from the analysis. Among 134 very preterm infants born between January 2014 and December 2020 at JBUH and discharged home, 93 were included in this study.

JBUH has a policy for the follow-up of very preterm infants. The preterm infant formula (16%), regular formula, and breast milk with fortifier provided in the NICU contained 0.24 mg, 0.66 mg, and 1.44 mg of iron per 100 mL, respectively. Iron supplementation (2.5 mg/kg/day with ferric hydroxide polymaltose complex containing 50 mg/mL of Fe^3+^) should be provided to very preterm patients at 2–4 weeks of age and maintained until at least 8 months regardless of their diet. The guardians were instructed to increase the amount of iron supplementation (2.5 mg/kg) according to the body weight of infants. Human milk fortifier or premature infant formula must be continued at least until full-term. Thereafter, the diet is provided based on the infant’s condition. Weaning should be started at the CA of 4–6 months and must begin with rice cereal and include iron-fortified food and beef to facilitate iron supplementation. For the first year, follow-up is performed at 4 (3–4), 8 (6–8), and 12 (10–12) months of CA. At each follow-up, the patient’s length, weight, and head circumference growth are measured. At birth, anthropometric parameters were measured at least twice by two nurses and measured again in the NICU if the obtained values differed by > 10%. The length and weight of infants visiting the outpatient clinic were measured with a length board using the gold standard method^8^. The infants also underwent physical and neurodevelopmental examinations. Developmental abnormalities in aspects such as motor, cognitive, and language skills were identified using a part of the Korean Infant Development Test^9^. Diet was also investigated to determine whether solid food including meat is started and whether iron supplementation according to weight (2.5 mg/kg) and cholecalciferol supplementation (400 IU/day) are adhered to. At 8 months of CA, blood test was also performed to confirm recovery from physiological anemia^10^. The examination included serum ferritin and iron levels, total iron binding capacity, calculated transferrin saturation (TS), and mean corpuscular volume (MCV). A retrospective study of patients discharged from the NICU based on the follow-up protocol of JBUH was conducted.

Weight, length, and head circumference data at birth and 8 months of CA were collected from the medical records. Z-scores of collected anthropometric data by GA were calculated based on the Intergrowth 21 chart^11^. Birth z-scores and corrected 8-month BMI were calculated by implementing BMI curves in preterm infants based on the American Academy of Pediatrics and the World Health Organization Growth Standard Chart, respectively^2,12^. SGA and extrauterine growth restriction (EUGR) were defined as below the 10th percentile at birth and 8 months of CA, respectively^13^. Rapid growths based on height, weight, head circumference, and BMI were defined as a z-score difference of > 1 standard deviation (SD) between birth and 8 months with consideration of catch-up growth^6,14,15^. ID was defined by two or more of the following parameters: ferritin level < 12 mcg/L, MCV < 71 fL, and TS < 10%. Anemia was defined as a hemoglobin level of < 10.5 g/dL at 8 months of CA^16^. IDA was diagnosed when both ID and anemia criteria were met.

The perinatal characteristics of preterm infants on NICU admission and their status at discharge were collected. The perinatal characteristics included Apgar score, GA, sex, and neonatal and maternal disease. The diseases observed in premature infants during NICU admission included respiratory distress syndrome (RDS), intraventricular hemorrhage, necrotizing enterocolitis, bronchopulmonary dysplasia, duration of ventilation, sepsis, and retinopathy of prematurity. The maternal diseases associated with anemia included pregnancy-induced hypertension, maternal diabetes mellitus, and abnormal placenta. Data on hemoglobin levels and MCV at discharge were also collected. Further, information on abnormal neurodevelopment, feeding type, and solid food initiation at 8 months of CA was obtained. Feeding types were classified into exclusive breastfeeding, formula feeding, and mixed feeding.

The exposure and outcome were Rapid BMI Increase (RBI) and ID at 8 months of CA, respectively. The very preterm infants included in the study were divided into two groups according to RBI. The values measured at birth and 8 months of CA and iron status from blood tests at 8 months of CA were compared. Continuous variables are reported as the mean and standard deviation. Categorical variables are reported as frequencies and percentages. Demographic factors and outcomes between groups were compared using the analysis of variance. The χ^2^ or Fisher’s exact tests for proportions were performed. A logistic regression model was used to determine the risk factors for ID at a CA of 8 months. GA and other factors with statistical significance based on ID were included as variables in the multivariate regression analysis with backward stepwise variable selection. The risk factors for the outcomes were evaluated based on the odd ratios and confidence intervals. All statistical analyses were conducted using R version 4.1.1 (R Foundation for Statistical Computing, Vienna, Austria). P-values of < 0.05 were considered statistically significant.

### Ethics declarations

This study was approved by the Institutional Review Board of Jeonbuk National University Hospital (IRB No. 2016-07-018-002) and conducted following the Declaration of Helsinki. The need for informed consent was waived by the ethics committee/institutional review board of Jeonbuk National University Hospital.

## Results

Among the 41 patients who were excluded, 17 were lost during follow-up, and 12 did not have data on physical measurements at the CA of 8 months due to early follow-up. The remaining 12 patients were excluded from the analysis due to blood test refusal. All patients underwent blood tests with iron profile and CRP levels, which are used to diagnose ID.

**Figure 1:**
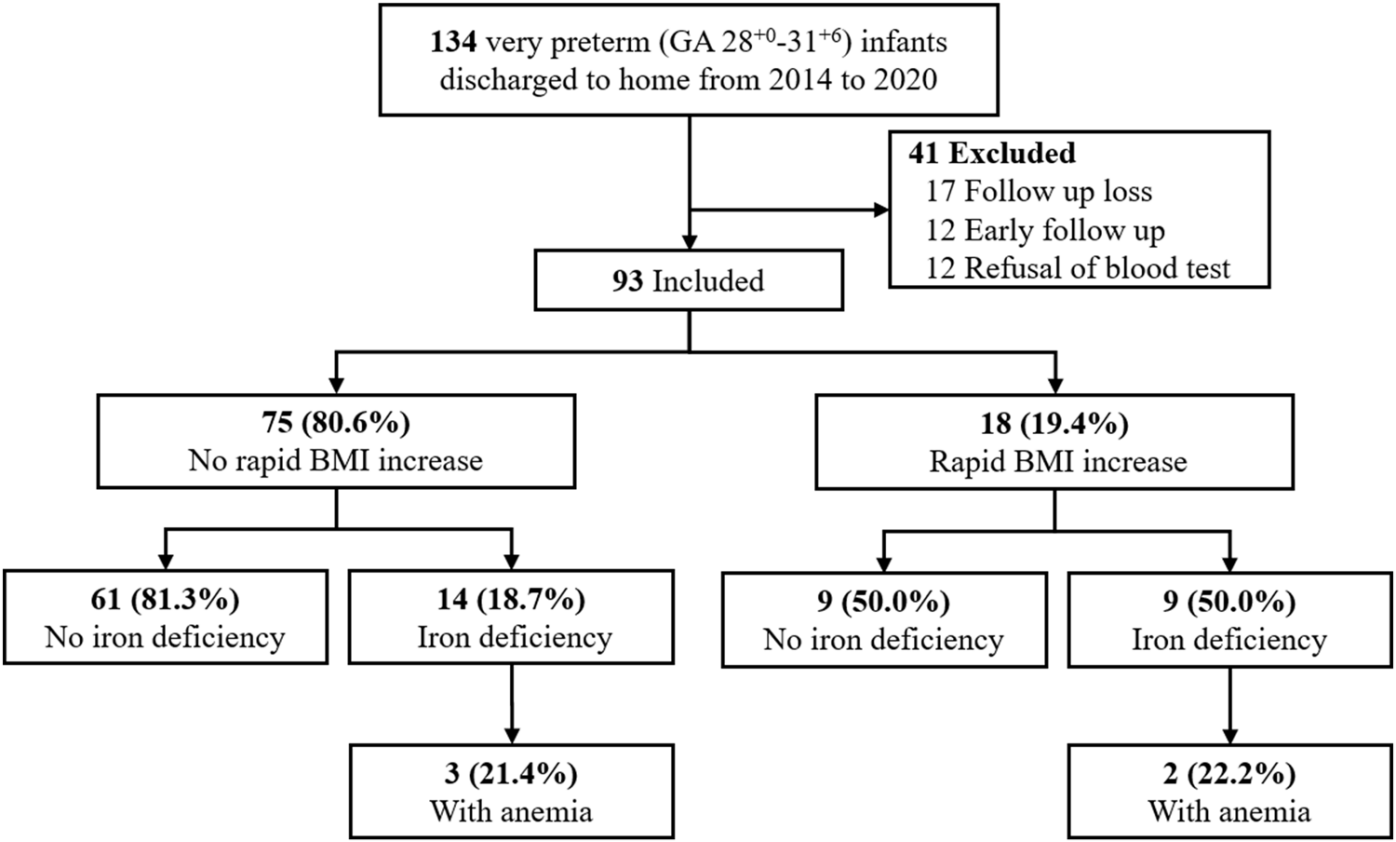
Flow chart showing the study population selection. All infants with normal iron status showed normal hemoglobin ranges.

The mean GA and birth weight of 93 very preterm infants included in the study were 30.1 ± 1.1 weeks and 1419.1 ± 291.5 g, respectively. A total of 41 patients were excluded from the study, and there was no difference in gestational age and birth weight between the included (30.4 ± 1.1 weeks, 1488.5 ± 291.2 g) and the excluded group (P-value of 0.19 and 0.21). At 8 months of CA, IDA and ID occurred in 5 of 93 (5.4%) and 23 of 93 (24.7%) infants, respectively. Between birth and 8 months of CA, 18 (19.4%) infants showed RBI, of which 9 (50%) had concurrent ID. Among 75 (80.6%) infants in the non-RBI group, 14 (18.7%) had ID. Anemia at 8 months of CA was not present in very preterm infants with normal iron status. All infants with anemia were confirmed to have IDA (Fig. 1).

The clinical characteristics of very preterm infants were compared according to RBI. The group with RBI had a greater GA and a higher incidence of RDS. Except for these differences, perinatal characteristics, morbidity, and duration of NICU admission did not differ significantly according to RBI. All infants were discharged without any respiratory or dietary assistance. There was no significant difference between the two groups in terms of hemoglobin levels, MCV, and the incidence rate of anemia. All infants survived up to 8 months of CA. No difference was found in terms of neurodevelopment and feeding type at the 8-month follow-up visit (Table 1). The clinical characteristics of very preterm infants when compared according to ID at 8 months of CA showed that the ID group had a higher proportion of patients with small gestational age (SGA). However, there was no significant difference in terms of other characteristics (Supplementary Table S1).

**Table 1:** Clinical characteristics and neonatal morbidities.

	All cohort (N = 93)	No rapidly increased BMI (N = 75)	Rapidly increased BMI (N = 18)	*P-*value
Perinatal demographic characteristics				
Male, n (%)	48 (51.6%)	38 (50.7%)	10 (55.6%)	0.91
Gestational age, (week)	30.1 ± 1.1	30.0 ± 1.0	30.5 ± 1.1	< 0.05
Vaginal delivery, n (%)	19 (20.4%)	18 (24.00%)	1 (5.6%)	0.16
Multiple, n (%)	25 (26.9%)	17 (22.67%)	8 (44.4%)	0.12
Apgar score at 1 min	6.1 ± 1.3	6.1 ± 1.2	6.1 ± 1.4	0.98
Apgar score at 5 min	7.61 ± 1.16	7.6 ± 1.2	7.8 ± 1.2	0.51
Small for gestational age, n (%)	10 (10.8%)	6 (8.0%)	4 (22.2%)	0.19
Maternal hypertension, n (%)	18 (19.35%)	13 (17.33%)	5 (27.78%)	0.50
Maternal diabetes mellitus, n (%)	8 (8.60%)	8 (10.67%)	0 ( 0.0%)	0.33
Abnormal placenta, n (%)	6 (6.45%)	5 (6.67%)	1 (5.56%)	1.00
Neonatal morbidities in the NICU				
RDS, n (%)	83 (89.3%)	70 (93.3%)	13 (72.2%)	< 0.05
IVH grade 3–4, n (%)	1 (1.1%)	1 (1.3%)	0 (0.0%)	1.00
NEC grade 2b–3, n (%)	1 (1.1%)	1 (1.3%)	0 (0.0%)	1.00
BPD moderate to severe, n (%)	3 (3.2%)	3 (4.0%)	0 (0.0%)	0.91
Duration of ventilation, days	9.8 ± 12.1	10.5 ± 12.7	7.2 ± 9.1	0.30
Sepsis, n (%)	4 (4.3%)	4 (5.3%)	0 (0.0%)	0.72
ROP stages 3–4, n (%)	13 (14.0%)	12 (16.0%)	1 (5.6%)	0.44
Packed RBC transfusion during admission, n (%)	18 (19.4%)	15 (20.0%)	3 (16.7%)	1.00
NICU hospital day, day	45.1 ± 19.2	44.7 ± 15.1	46.6 ± 28.8	0.76
Clinical characteristics between discharge and 8 months				
Corrected age at discharge, weeks	36.5 ± 2.3	36.6 ± 2.4	36.5 ± 1.9	0.91
Hemoglobin at discharge, g/dL	10.4 ± 1.8	10.4 ± 1.8	10.6 ± 2.2	0.62
MCV at discharge, fL	93.8 ± 5.5	93.4 ± 5.6	95.8 ± 4.7	0.09
Abnormal neurodevelopment at 8 months				
Motor, n (%)	11 (11.8%)	10 (13. 3%)	1 (5.6%)	0.61
Cognition, n (%)	4 (4.3%)	4 (5.3%)	0 (0.0%)	0.72
Language, n (%)	10 (11.0%)	8 (11.0%)	2 (11.1%)	1.00
Corrected age at 8 months follow-up, months	7.0 ± 0.5	7.0 ± 0.5	7.0 ± 0.5	0.79
Extrauterine growth restriction, n (%)	7 (7.5%)	6 (8.0%)	1 (5.6%)	1.00
Readmission after discharge, n (%)	38 (40.9%)	30 (40.0%)	8 (44.4%)	0.94
Feeding type at 8 months				
Exclusive breastfeeding, n (%)	17 (18.3%)	14 (18.7%)	3 (16.7%)	1.00
Exclusive formula feeding, n (%)	61 (65.6%)	50 (66.7%)	11 (61.1%)	0.87
Mixed feeding, n (%)	7 (7.5%)	6 (8.0%)	1 (5.6%)	1.00
Initiation of solid food, n (%)	81 (87.1%)	66 (88.0%)	15 (83.3%)	0.89
Death after discharge, n (%)	0 (0.0%)	0 (0.0%)	0 (0.0%)	-

Data are shown as N (%) or mean ± standard deviation.

*BPD* bronchopulmonary dysplasia, *IVH* intraventricular hemorrhage, *NEC* necrotizing enterocolitis, *NICU* neonatal intensive care unit, *RDS* respiratory distress syndrome, *ROP* retinopathy of premature.

No difference in length, weight, or head circumference at birth was found between the RBI and non-RBI groups. BMI at birth was lower in the RBI group than in the non-RBI group. At 8 months of CA, no difference was noted in the head circumference or length. However, the RBI group weighed more than the non-RBI group. When differences in z-scores between birth and 8 months of CA were evaluated, a difference in weight was found. When changes in z-scores between birth and 8 months of CA were compared according to RBI, a statistically significant difference in weight but not in length was noted. Ferritin, MCV, serum iron, and transferrin saturation values indicating iron status at 8 months of CA were generally lower in the RBI group than the non-RBI group, although not significantly lower. However, when ID was classified by applying diagnostic criteria, more infants with RBI were diagnosed with ID than those without RBI. No difference was found in the frequency of anemia according to RBI. Then, anthropometric data of very premature infants were compared according to ID at 8 months of CA. No significant difference was found in length, weight, head circumference, or BMI at birth or 8 months of CA (Table 2, Supplemental Table S2).

**Table 2:** Anthropometric data and iron status at birth and 8 months of corrected age.

	All cohort (N = 93)	No rapid increase in BMI(N = 75)	Rapid increase in BMI(N = 18)	*P-*value	No iron deficiency (N = 70)	Iron deficiency (N = 23)	*P-*value
Anthropometric data at birth and 8 months of CA							
Length at birth, cm	38.9 ± 2.7	38.7 ± 2.7	39.4 ± 2.6	0.31	38.9 ± 2.6	38.7 ± 2.9	0.81
Weight at birth, g	1419.1 ± 291.5	1434.3 ± 289.1	1356.1 ± 301.4	0.31	1424.9 ± 286.9	1401.7 ± 311.2	0.74
Head circumference at birth, cm	27.5 ± 2.0	27.5 ± 2.0	27.5 ± 1.9	0.93	27.4 ± 2.0	27.8 ± 1.9	0.39
BMI at birth, kg/m^2^	9.3 ± 1.2	9.5 ± 1.1	8.6 ± 1.1	< 0.05	9.3 ± 1.1	9.2 ± 1.2	0.69
Length at 8 months, cm	67.7 ± 3.2	67.8 ± 3.2	67.0 ± 3.5	0.36	67.7 ± 3.1	67.4 ± 3.7	0.70
Weight at 8 months, kg	8.2 ± 1.2	8.1 ± 1.2	8.9 ± 1.2	< 0.05	8.2 ± 1.1	8.4 ± 1.4	0.41
Head circumference at 8 months, cm	43.0 ± 1.7	42.9 ± 1.7	43.2 ± 1.7	0.50	43.0 ± 1.6	43.0 ± 2.0	0.94
BMI at 8 months, kg/m^2^	17.9 ± 1.8	17.5 ± 1.6	19.7 ± 1.7	< 0.05	17.7 ± 1.8	18.4 ± 1.8	0.17
Rapid BMI growth by z-score n (%)	18 (19.4%)	–	–	–	9 (12.9%)	9 (39.1%)	< 0.05
Rapid length growth by z-score n (%)	50 (53.8%)	42 (56.0%)	8 (44.4%)	0.43	38 (54.3%)	12 (52.2%)	1.00
Rapid weight growth by z-score n (%)	30 (32.3%)	13 (17.3%)	17 (94.4%)	< 0.05	18 (25.7%)	12 (52.2%)	< 0.05
Rapid head circumference growth by z-score n (%)	25 (26.9%)	18 (24.0%)	7 (38.9%)	0.33	19 (27.1%)	6 (26.1%)	1.00
Iron status at 8 months of CA							
Hemoglobin, g/dL	12.5 ± 1.0	12.5 ± 0.1	12.5 ± 1.2	0.96	12.77 ± 0.81	11.75 ± 1.20	< 0.05
Ferritin, mcg/L	24.4 ± 15.9	25.5 ± 16.2	20.0 ± 14.2	0.19	27.9 ± 15.0	13.9 ± 14.3	< 0.05
MCV, fL	73.6 ± 3.3	73.7 ± 3.3	73.1 ± 3.1	0.45	74.3 ± 3.1	71.5 ± 2.9	< 0.05
Serum iron, micromol/L	68.6 ± 28.5	68.6 ± 8.9	68.9 ± 27.8	0.97	73.2 ± 29.0	54.7 ± 22.4	< 0.05
Transferrin saturation, %	16.3 ± 8.9	16.5 ± 9.2	15.4 ± 7.7	0.67	18.0 ± 9.0	11.1 ± 6.1	< 0.05
Iron deficiency, n (%)	23 (24.7%)	14 (18.7%)	9 (50.0%)	< 0.05	−	−	−
Iron deficiency anemia, n (%)	5 (5.4%)	3 (4.0%)	2 (11.1%)	0.54	0 (0.0%)	5 (21.7%)	< 0.05

Data are shown as N (%) or mean ± standard deviation.

*BMI* body mass index, *CA* corrected age, *MCV* mean cell volume.

Logistic regression analysis was performed to identify the risk factors of ID at 8 months of CA. The ID group had more infants with RBI and SGA than those without ID. EUGR, feeding type, and solid food initiation at 8 months of CA did not differ significantly according to ID (Supplemental Table S1). In the univariate logistic regression analysis, SGA, rapid weight growth by z-score, and RBI by z-score, which showed statistical differences, were identified as significant factors. In the multivariate logistic regression controlling for GA, SGA (odds ratio [OR] 6.06, 95% confidence interval [CI] 1.34–30.21) and RBI by z-score (OR 4.26, 95% CI 1.28–14.65) were identified as independent risk factors for ID (Table 3).

**Table 3:** Risk factor for iron deficiency at corrected age of 8 months.

	Univariate				Multivariate			
	OR	95% CI	95% CI	*P* value	OR	95% CI	95% CI	*P* value
Gestational age, (week)	1.32	0.84	2.09	0.232				
Apgar score at 1 min	1.47	1.00	2.23	0.054				
Small for gestational age, n (%)	5.82	1.50	25.06	0.012	6.06	1.34	30.21	0.020
Rapid weight growth by z-score	3.15	1.19	8.53	0.021				
Rapid BMI growth by z-score	4.36	1.46	13.25	0.008	4.26	1.28	14.65	0.018

*BMI* body mass index, *CI* confidence interval.

## Discussion

RBI between birth and 8 months of CA was identified as a risk factor for ID at 8 months of CA. Among the criteria for rapid growth in very preterm infants, RBI was found to be an independent risk factor for ID. Since RBI is the catch-up in the growth of very preterm infants, it may result from a rapid increase in relative weight without maintaining an overall balance in growth. The mean iron requirements for growth include blood volume expansion and body mass gain. As the infant grows and blood volume expands, hemoglobin requires increased amounts of iron^17^. ID at infancy was associated with a faster growth between 0 and 12 months^18–20^. Inappropriate provision of iron supplementation according to the rapidly increasing weight of infants may also increase the risk of ID.

In this study, ID/IDA occurred in 24.7%/5.4% of very preterm infants. In a prospective study in Brazil targeting a similar cohort, the rates of anemia and ID in very LBW infants at 1 year of CA were 26.4% and 48.0%, respectively^1^. Prophylactic iron supplementation in premature infants reduced the prevalence of anemia at 12 months of CA^21^. Adequate iron supplementation was ensured via follow-up during infancy among very preterm infants. Iron supplementation at 2 mg/kg/day until 6 months of life in marginally LBW infants is an effective intervention to prevent early IDA^12^. In a recent retrospective study of very preterm infants, early iron prophylaxis was performed at 2–4 weeks of chronological age. However, ID was confirmed in 32% of very preterm infants before 6 months of CA^22^. Considering that a similar rate of ID was also observed in this study, very preterm infants require a higher iron supply to prevent ID compared with term or moderate preterm infants.

Reticulocyte hemoglobin levels were used to differentiate ID from other causes of anemia^23^. Iron sufficiency measured based on reticulocyte hemoglobin levels at discharge from the NICU was confirmed to be 20% and increased to 39% after educational efforts and standardized guidelines were applied^24^. Although the reticulocyte hemoglobin level was not measured in this study, with consideration of hemoglobin levels at discharge, iron supplementation is required from the early period of birth.

SGA in very preterm infants was also considered a risk factor of ID at 8 months of CA. In a recent prospective study of neonates under 33 weeks of age or those with SGA, ID was confirmed in the cord blood immediately after birth in 16% of patients and at 1 month of age in 32%^25^. Since ID is common in late preterm infants at approximately 6 weeks of age, low birth weight and low serum ferritin levels within the first week of life are associated with a high risk of iron depletion, requiring iron supplementation in the early stages of life^26^. A previous study on late preterm infants also revealed that infants with SGA had lower ferritin levels and total body iron stores^27^. Thus, neonates with SGA require a large amount of iron supply.

After birth, the rapid catch-up in weight and head circumference of premature infants has a positive effect on neurocognitive prognosis. Pediatricians try to keep the growth as fast as possible because the association among later adiposity, insulin resistance, and cardiovascular diseases, which can result from rapid weight growth, is unknown^5^. However, when normal-weight children gain weight rapidly, they have an increased risk of becoming overweight and obese rather than developing skeletal maturity^7^. In a single center study in which 834 premature infants were followed-up for 1 year^21^. A rapid increase in BMI in SGA newborns can increase the risk of persistent obesity^14^. The optimal pattern for infant weight growth is thought to be different among populations^28^. Therefore, the criteria for rapid growth that can lead to a worse poor prognosis must be presented.

Rapid growth in the neonatal period has different definitions, and the relative z-score is mainly used. Stetter defined rapid growth in the neonatal period as an increase in the z-score of ≥ 1 SD from birth to 4 months of age and suggested that meeting this criterion as a newborn is associated with obesity as a child and young adult^6^. Thus far, the concept of growth in premature infants has mainly focused on comparing outcomes according to whether growth is slow^5^. Considering the catch-up growth of very preterm infants, setting the standard for rapid growth in infancy after birth to when the z-score has increased by ≥ 1 SD among various criteria appears reasonable. Previous studies of full-term children have reported rapid growth in 21.8% and 28.7%^6,7^. In this study, the population of premature infants was small, which could have been due to the set conservative standard for catch-up growth in very preterm infants. Because the catch-up growth of very preterm infants should be a range of growth in infants without diseases, the BMI presented in this study can be considered the upper limit of catch-up growth.

This study had some limitations. First, the ID risk factor analysis could not include compliance with recommended iron supplementation amounts, which should be checked through follow-up observations at 4 and 8 months of CA. Moreover, statistical analysis was difficult because of the subjectivity of parents’ answers rather than quantitative indicators. Second, the iron status at birth could not be confirmed. Considering the relationship between SGA and iron status at birth reported in previous studies, ID due to decreased iron storage was reflected in SGA infants. Third, the TfR was not included in the previously presented diagnostic criteria of ID because it could not be tested in our hospital. Although ID was judged to be underestimated, its prevalence being similar to its diagnostic criteria indicated that it might have a small effect on the results of this study. Finally, data were insufficient data, including dietary data between discharge and 8 months of CA, because of the retrospective design.

The rapid growth of premature infants is thought to have a similar effect. Adequate iron supply must be maintained to prevent ID in premature infants, which were previously identified as causes of ID and IDA^29^.

## Conclusion

In this study, the RBI of very preterm infants in early life and SGA were risk factors for ID at 8 months of CA. Consequently, it underscores the need for tailored nutritional strategies for this vulnerable subgroup. Further prospective studies of ID based on proper BMI increases of very preterm infants are needed.

### Supplementary Information

Supplementary Table 1.

Supplementary Table 2.

## Data Availability

Additional anonymized data are available from the corresponding authors upon reasonable request.

## References

[1] Ferri C, Procianoy RS, Silveira RC (2014). Prevalence and risk factors for iron-deficiency anemia in very-low-birth-weight preterm infants at 1 year of corrected age. J. Trop. Pediatr..

[2] Domellof M (2014). Iron requirements of infants and toddlers. J. Pediatr. Gastroenterol. Nutr..

[3] Akman M (2004). The effects of iron deficiency on infants' developmental test performance. Acta Paediatr..

[4] Berglund S, Westrup B, Domellof M (2010). Iron supplements reduce the risk of iron deficiency anemia in marginally low birth weight infants. Pediatrics.

[5] Ong KK (2015). Postnatal growth in preterm infants and later health outcomes: A systematic review. Acta Paediatr..

[6] Stettler N, Kumanyika SK, Katz SH, Zemel BS, Stallings VA (2003). Rapid weight gain during infancy and obesity in young adulthood in a cohort of African Americans. Am. J. Clin. Nutr..

[7] Cameron N, Pettifor J, De Wet T, Norris S (2003). The relationship of rapid weight gain in infancy to obesity and skeletal maturity in childhood. Obes. Res..

[8] Wood AJ, Raynes-Greenow CH, Carberry AE, Jeffery HE (2013). Neonatal length inaccuracies in clinical practice and related percentile discrepancies detected by a simple length-board. J. Paediatr. Child. Health.

[9] Kim D (2023). Korean Developmental Screening Test for Infants and Children (K-DST): Development, applications, and implications for future early childhood development interventions. Clin. Exp. Pediatr..

[10] Widness JA (2008). Pathophysiology of anemia during the neonatal period, including anemia of prematurity. NeoReviews.

[11] Villar J (2016). INTERGROWTH-21st very preterm size at birth reference charts. Lancet.

[12] Berglund SK, Westrup B, Domellof M (2015). Iron supplementation until 6 months protects marginally low-birth-weight infants from iron deficiency during their first year of life. J. Pediatr. Gastroenterol. Nutr..

[13] Peila C (2020). Extrauterine growth restriction: Definitions and predictability of outcomes in a cohort of very low birth weight infants or preterm neonates. Nutrients.

[14] Wu D (2021). Rapid BMI increases and persistent obesity in small-for-gestational-age infants. Front. Pediatr..

[15] Nash A (2011). Pattern of growth of very low birth weight preterm infants, assessed using the WHO Growth Standards, is associated with neurodevelopment. Appl. Physiol. Nutr. Metab..

[16] Berglund SK, Westrup B, Hagglof B, Hernell O, Domellof M (2013). Effects of iron supplementation of LBW infants on cognition and behavior at 3 years. Pediatrics.

[17] Chaparro CM (2008). Setting the stage for child health and development: Prevention of iron deficiency in early infancy. J. Nutr..

[18] Domellof M (2017). Meeting the iron needs of low and very low birth weight infants. Ann. Nutr. Metab..

[19] Thorsdottir I, Gunnarsson BS, Atladottir H, Michaelsen KF, Palsson G (2003). Iron status at 12 months of age: Effects of body size, growth and diet in a population with high birth weight. Eur. J. Clin. Nutr..

[20] Gunnarsson BS, Thorsdottir I, Palsson G (2004). Iron status in 2-year-old Icelandic children and associations with dietary intake and growth. Eur. J. Clin. Nutr..

[21] Deng Y, Yang F, Mu D (2019). First-year growth of 834 preterm infants in a Chinese population: A single-center study. BMC Pediatr..

[22] Landry C (2022). Postdischarge iron status in very preterm infants receiving prophylactic iron supplementation after birth. J. Pediatr..

[23] Mast AE, Blinder MA, Dietzen DJ (2008). Reticulocyte hemoglobin content. Am. J. Hematol..

[24] Morton SU (2020). Screening with reticulocyte hemoglobin increased iron sufficiency among NICU patients. Pediatr. Qual. Saf..

[25] Brichta CE, Godwin J, Norlin S, Kling PJ (2022). Impact and interactions between risk factors on the iron status of at-risk neonates. J. Perinatol..

[26] Akkermans MD (2016). Predictive factors of iron depletion in late preterm infants at the postnatal age of 6 weeks. Eur. J. Clin. Nutr..

[27] Kim HA, Park SH, Lee EJ (2019). Iron status in small for gestational age and appropriate for gestational age infants at birth. Korean J. Pediatr..

[28] Singhal A (2017). Long-term adverse effects of early growth acceleration or catch-up growth. Ann. Nutr. Metab..

[29] Ziegler EE (2011). Consumption of cow's milk as a cause of iron deficiency in infants and toddlers. Nutr. Rev..

