# Supplementary material for: Rapid increase in the body mass index of very preterm infants is a risk factor for iron deficiency during infancy
Source: Sci Rep. 2023 Sep 19;13:15526. doi: 10.1038/s41598-023-42531-1 (PMC10509161; doi:10.1038/s41598-023-42531-1)
Supplement: Supplementary file 1 — Supplementary Table 1. [file 41598_2023_42531_MOESM1_ESM.docx]

**Supplement Table S1. Clinical characteristics and neonatal morbidities**

|  | **No iron deficiency**  **(N = 70)** | **Iron deficiency**  **(N = 23)** | ***P-*value** |
| --- | --- | --- | --- |
| **Perinatal demographic characteristics** |  |  |  |
| Male, n (%) | 33 (47.1%) | 15 (65.2%) | 0.21 |
| Gestational age, (week) | 30.0 ± 1.1 | 30.3 ± 0.9 | 0.23 |
| Vaginal delivery, n (%) | 16 (22.9%) | 3 (13.0%) | 0.48 |
| Multiple, n (%) | 19 (27.1%) | 6 (26.1%) | 1.00 |
| Apgar score at 1 min | 6.0 ± 1.2 | 6.6 ± 1.4 | 0.05 |
| Apgar score at 5 min | 7.5 ± 1.1 | 7.9 ± 1.3 | 0.43 |
| Small for gestational age, n (%) | 4 (5.7%) | 6 (26.1%) | <0.05 |
| Maternal hypertension, n (%) | 12 (17.1%) | 6 (26.1%) | 0.52 |
| Maternal diabetes mellitus, n (%) | 7 (10.0%) | 1 (4.4%) | 0.68 |
| Abnormal placenta, n (%) | 5 (6.7%) | 1 (5.6%) | 1.00 |
| **Neonatal morbidities in the NICU** |  |  |  |
| RDS, n (%) | 64 (91.4%) | 19 (82.6%) | 0.43 |
| IVH grades 3–4, n (%) | 1 (1.3%) | 0 (0.0%) | 1.00 |
| NEC grades 2b–3, n (%) | 1 (1.4%) | 0 (0.0%) | 1.00 |
| BPD moderate to severe, n (%) | 2 (2.9%) | 1 (4.4%) | 1.00 |
| Duration of ventilation, days | 9.47 ± 11.90 | 10.87 ± 12.82 | 0.63 |
| Sepsis, n (%) | 3 (4.3%) | 1 (4.4%) | 1.00 |
| ROP stages 3–4, n (%) | 10 (14.3%) | 3 (13.0%) | 1.00 |
| Transfusion during admission, n (%) | 16 (22.9%) | 2 (8.7%) | 0.24 |
| NICU hospital day, day | 46.0 ± 19.3 | 41.7 ± 19.1 | 0.40 |
| **Clinical characteristics between discharge and 8 months** |  |  |  |
| Corrected age at discharge, weeks | 36.4 ± 1.6 | 37.0 ± 3.8 | 0.49 |
| Hemoglobin at discharge, g/dL | 10.5 ± 1.8 | 10.4 ± 1.8 | 0.87 |
| MCV at discharge, fL | 93.9 ± 5.7 | 93.7 ± 5.1 | 0.90 |
| Abnormal neurodevelopment at 8 months |  |  |  |
| Motor, n (%) | 11 (15.7%) | 0 (0.0%) | 0.10 |
| Cognition, n (%) | 3 (4.3%) | 1 (4.4%) | 1.00 |
| Language, n (%) | 8 (11.0%) | 2 (11.1%) | 1.00 |
| Corrected age at 8 months follow-up, weeks | 7.0 ± 0.5 | 7.1 ± 0.4 | 0.49 |
| Extrauterine growth restriction, n (%) | 4 (5.7%) | 3 (13.0%) | 0.48 |
| Readmission after discharge, n (%) | 28 (40.0%) | 10 (43.5%) | 0.96 |
| Feeding type at 8 months |  |  |  |
| Exclusive breastfeeding, n (%) | 12 (17.1%) | 5 (21.7%) | 0.86 |
| Exclusive formula feeding, n (%) | 46 (65.7%) | 15 (65.2%) | 1.00 |
| Mixed feeding, n (%) | 6 (8.6%) | 1 (4.4%) | 0.83 |
| Initiation of weaning food, n (%) | 60 (85.7%) | 21 (91.3%) | 0.74 |
| Death after discharge, n (%) | 0 (0.0%) | 0 (0.0%) | - |

BPD, bronchopulmonary dysplasia; IVH, intraventricular hemorrhage; NEC, necrotizing enterocolitis; NICU, neonatal intensive care unit; RDS, respiratory distress syndrome; ROP, retinopathy of premature. Data are shown as N (%) or mean ± standard deviation.
