# Supplementary material for: Rapid increase in the body mass index of very preterm infants is a risk factor for iron deficiency during infancy
Source: Sci Rep. 2023 Sep 19;13:15526. doi: 10.1038/s41598-023-42531-1 (PMC10509161; doi:10.1038/s41598-023-42531-1)
Supplement: Supplementary file 2 — Supplementary Table 2. [file 41598_2023_42531_MOESM2_ESM.docx]

**Supplement Table S2. Anthropometric data and iron status at birth and 8 months of CA by z-score**

|  | **All cohort**  **(N = 93)** | **No rapid**  **increase in BMI**  **(N = 75)** | **Rapid**  **Increase in BMI**  **(N = 18)** | ***P-*value** | **No iron deficiency**  **(N = 70)** | **Iron deficiency**  **(N = 23)** | ***P-*value** |
| --- | --- | --- | --- | --- | --- | --- | --- |
| Length at birth, z-score | −0.32 ± 0.78 | −0.31 ± 0.79 | −0.34 ± 0.76 | 0.88 | −0.26 ± 0.73 | −0.49 ± 0.93 | 0.22 |
| Weight at birth, z-score | 0.13 ± 0.91 | 0.25 ± 0.88 | −0.39 ± 0.88 | <0.05 | 0.21 ± 0.84 | −0.12 ± 1.08 | 0.14 |
| Head circumference at birth, z-score | −0.11 ± 1.01 | −0.05 ± 1.05 | −0.34 ± 0.80 | 0.28 | −0.12 ± 1.02 | −0.06 ± 1.02 | 0.78 |
| BMI at birth, z-score | 0.58 ± 1.10 | 0.80 ± 1.03 | −0.37 ± 0.89 | <0.05 | 0.65 ± 1.08 | 0.35 ± 1.16 | 0.25 |
| Length at 8 months, z-score | 0.72 ± 1.25 | 0.79 ± 1.24 | 0.44 ± 1.28 | 0.29 | 0.81 ± 1.19 | 0.46 ± 1.41 | 0.25 |
| Weight at 8 months, z-score | 0.65 ± 1.05 | 0.51 ± 1.03 | 1.24 ± 0.91 | <0.05 | 0.64 ± 0.99 | 0.70 ± 1.23 | 0.82 |
| Head circumference at 8 months, z-score | 0.23 ± 1.24 | 0.19 ± 1.26 | 0.44 ± 1.16 | 0.45 | 0.27 ± 1.16 | 0.13 ± 1.46 | 0.66 |
| BMI at 8 months, z-score | 0.47 ± 1.17 | 0.20 ± 1.05 | 1.62 ± 0.93 | <0.05 | 0.39 ± 1.15 | 0.72 ± 1.20 | 0.24 |
| Length difference, z-score | 1.04 ± 1.12 | 1.10 ± 1.08 | 0.78 ± 1.27 | 0.281 | 1.07 ± 1.12 | 0.95 ± 1.15 | 0.67 |
| Weight difference, z-score | 0.53 ± 0.97 | 0.26 ± 0.83 | 1.63 ± 0.71 | <0.05 | 0.43 ± 0.92 | 0.82 ± 1.10 | 0.10 |
| Head circumference difference, z-score | 0.34 ± 1.07 | 0.24 ± 1.12 | 0.77 ± 0.70 | <0.05 | 0.39 ± 1.09 | 0.19 ± 1.01 | 0.44 |
| BMI difference, z-score | −0.10 ± 1.35 | −0.6 ± 0.9 | 2.0 ± 0.5 | <0.05 | −0.26 ± 1.28 | 0.38 ± 1.47 | <0.05 |

BMI, body mass index; CA, corrected age. Data are shown as N (%) or mean ± standard deviation
